# The Probiotic Strain *Lactobacillus acidophilus* CL1285 Reduces Fat Deposition and Oxidative Stress and Increases Lifespan in *Caenorhabditis elegans*

**DOI:** 10.3390/microorganisms12061036

**Published:** 2024-05-21

**Authors:** Samir Bouasker, Sonja Nodland, Mathieu Millette

**Affiliations:** 1 Bio-K+, a Kerry Company, 495 Boulevard Armand-Frappier, Laval, QC H7V 4B3, Canada; mathieu.millette@kerry.com; 2 Kerry, 3400 Millington Rd, Beloit, WI 53511, USA; sonja.nodland@kerry.com

**Keywords:** probiotics, *L. acidophilus* CL1285, *C. elegans*, fat accumulation, *daf-16*, life extension, glucose, Nile Red, Oil Red

## Abstract

*Caenorhabditis elegans* was recently shown to be a powerful model for studying and identifying probiotics with specific functions. *Lactobacillus acidophilus* CL1285, *Lacticaseibacillus casei* LBC80R, and *Lacticaseibacillus rhamnosus* CLR2, which are three bacteria that were marketed by Bio-K+, were evaluated using the nematode *C. elegans* to study fat accumulation, lifespan, and resistance to oxidative stress. Although the general effects of probiotics in terms of protection against oxidative stress were highlighted, the CL1285 strain had an interesting and specific feature, namely its ability to prevent fat accumulation in nematodes; this effect was verified by both the Oil Red and Nile Red methods. This observed phenotype requires daf-16 and is affected by glucose levels. In addition, in a daf-16- and glucose-dependent manner, CL1285 extended the lifespan of *C. elegans*; this effect was unique to CL1285 and not found in the other *L. acidophilus* subtypes in this study. Our findings indicate that *L. acidophilus* CL1285 impacts fat/glucose metabolism in *C. elegans* and provides a basis to further study this probiotic, which could have potential health benefits in humans and/or in mammals.

## 1. Introduction

The gut microbiota comprise 10^14^ microorganisms that are specifically adapted to this particular environment and include bacteria, viruses, parasites, and fungi with mutualistic interactions [1]. Distributed in the small intestine and the colon, the microbiota create a protective ecosystem between the lumen and mucus layer, which is relevant for many biological functions and plays a central role in human health. For example, bacterial enzymes not encoded by the human genome are necessary as they produce compounds that are essential for life. Microbiota contribute to the synthesis of many essential molecules, such as short-chain fatty acids (SCFAs) and vitamins, via the digestion of complex substrates [2]. Moreover, gut bacteria modulate energy obtained from the diet and alterations of the microbiota; for example, changes in the ratio between Firmicutes and Bacteroidetes are often linked to metabolic syndrome and obesity [3]. In addition, alteration in food intake by consumption of a Western-type diet and/or food lacking nondigestible fiber is linked to dysbiosis, low-grade inflammation, and endotoxemia due to LPS release, which results in the production of proinflammatory cytokines and the recruitment of immune cells to the intestinal tract [4]. The integrity of the intestinal barrier is subsequently affected, thus allowing for the establishment of chronic metabolic diseases, such as obesity or fat accumulation. Several examples of dysbiosis and metabolic disorders have been described [5,6]. Drug-induced disruption of the natural microbial balance by taking antibiotics, which are widely used in animal production, influences animal growth and fat metabolism [7]. More recently, the emergence of certain strains associated with health highlights the possibility of using bacteria for curative purposes, such as *Akkermansia muciniphila*, a mucin-degrading bacteria, whose abundance is correlated with obesity in humans and rodents [8,9]. Taken together, these data indicate that the modulation of the microbiota by dietary supplementation with probiotics/bacteria could be used for the prevention or mitigation of obesity or metabolic syndrome.

Probiotics are defined as live microorganisms that, when administered in adequate amounts, confer a health benefit to the host [10]. Since commercially available probiotics are of diverse bacterial genera and species, the quantities needed to confer health benefits vary and must be determined and proven by clinical trials. Concerning their safety, probiotics are considered harmless and are classified in the GRAS (generally recognized as safe) group by the FDA [11]. Originally, probiotic health benefits have been found to be restricted to the gastrointestinal tract and involve helping maintain a balanced and rich microbiota to protect against infection and the overgrowth of opportunistic pathogens [12]. Extensive in vitro research and clinical studies using mainly lactobacilli, bifidobacteria, and yeast species have clearly demonstrated protection from or a reduction in infection by intestinal pathogens such as *Helicobacter pylori* [13,14] and *Salmonella* [15,16,17]. These beneficial effects in prevention and/or treatment are not limited to the gastrointestinal tract, but similar beneficial effects have been reported for infections in the respiratory tract and bacterial vaginosis [18,19]. Direct inhibition of bacterial growth by probiotics, which secrete bacteriocins and organic acids, competition for substrates, and adhesion to the intestinal mucosa are the main known mechanisms of action [20]. However, the modulation of the immune system and the immune response by probiotics is an alternative and widespread mechanism that allows probiotics to act indirectly on various organs and provide protection throughout the body [21]. The probiotic combination of *Lactobacillus acidophilus* CL1285, *Lacticaseibacillus casei* LBC80R, and *Lacticaseibacillus rhamnosus* CLR2 prevented primary *Clostridioides difficile* infection in a randomized, double-blinded, placebo-controlled clinical study, and in vitro data suggested a direct inhibition mechanism [22,23]; however, the other potential beneficial effects of this formulation were not evaluated. Regarding obesity and metabolic syndrome, supplementation with some lactobacilli species has been shown to reduce weight gain in animal models of diet-induced obesity [24,25,26]. Clinical trial studies showing similar tendencies are also well documented; some of the effects included weight loss, reduced waist circumference, or lower levels of blood lipids [24,27,28,29,30,31]. *C. elegans* was recently identified as a valuable and efficient model for studying the effect of beneficial bacteria on probiotics, as several features of host–bacterial interactions are conserved between humans and nematodes [32]. Even if the composition of the gut microbiota of *C. elegans* is different, it remains an easy and straightforward model for studying putative probiotic benefits in terms of fat accumulation, oxidative stress, and aging in animals [33,34,35,36]. These three phenotypic markers are related, and excess fat is often associated with oxidative stress due to reactive oxygen species production, insulin pathway resistance, and metabolic disorders [37,38]. The advantage of worms is that they possess genes and metabolic pathways that are involved in lipid and glucose metabolism and are conserved and shared with humans, but they are also a unique model for dissecting the mechanisms underlying the beneficial properties of these metabolic pathways. Moreover, *C. elegans* can be used as a preclinical screening model for evaluating the effects of probiotics in various environmental conditions and with various genetic backgrounds.

In this paper, we describe the results of exposing *C. elegans* to Bio-K+ probiotic strains individually and highlight the resulting fat accumulation following the administration of one strain, *L. acidophilus* CL1285. In this study, we evaluated the effects of probiotic strains on fat accumulation and oxidative stress and explored the underlying mechanisms of these effects using high glucose exposure and a *daf-16* genetic background: the *daf-16* mutant alters insulin signaling and the IGF-1 signaling (IIS) pathway. Moreover, CL1285 was compared to other *L. acidophilus* strains, and the administration of CL1285 was found to have unique health benefits; thus, this strain should be used for further animal studies on weight regulation.

## 2. Materials and Methods

### 2.1. Bacteria/Probiotic Strains

*L. acidophilus* CL1285, *L. casei* LBC80R, and *L. rhamnosus* CLR2 are proprietary strains that were isolated from fecal samples. *L. acidophilus* strains NCFM, BP4981, and ATCC 314 were purchased from the *American Type Culture Collection* (ATCC, Manassas, VA, USA), the Belgian Coordinated Collections of Microorganisms (BCCM/LMG, Ghent, Belgium), and the Patent and Bio-Resource Centre (Chiba, Japan). Bacteria were stored at −80 °C and thawed at room temperature before use. Before each experiment, bacteria were streaked on Man, Rogosa, Sharpe (MRS) agar (EMDMillipore, Oakville, ON, Canada) plates to verify purity. All bacteria were grown on MRS broth (EMDMillipore, Oakville, ON, Canada) under an anaerobic atmosphere using a BD anaerobic container and BD GasPak EZ (BD, Mississauga, ON, Canada). After 3 days, the cultures were centrifuged and washed 3 times with M9 buffer (3 g KH_2_PO_4_, 6 g Na_2_HPO_4_, 5 g NaCl in 1 L H_2_O, 1 mL of 1 M MgSO_4_ after autoclaving 20 min 120 °C; Powder from Fischer Scientific, Ottawa, ON Canada). The pellet was then resuspended in M9 buffer at a concentration of 10 mg/mL, and 0.1 mL was seeded on the plate. OP50 strains (Escherichia coli OP50, Carolina, Burlington, NC, USA) were grown on Luria-Bertani (LB) media (Fisher, Fair Lawn, NJ, USA) and cultured overnight under aerobic conditions with shaking at 220 rpm at 37 °C (Thermo Scientific, Orbital Shakers-incubator, Waltham, MA, USA). The cultures were centrifuged and washed 3 times with M9 buffer; the pellet was then resuspended in M9 buffer at a concentration of 10 mg/mL, and 0.1 mL was seeded on the plate. OP50 strain is a reference strain used to maintain and work with nematodes. 

### 2.2. C. elegans Strains

The pmk-1 (km25) IV, daf-2 (e1370) III, and daf-16 (mgDf50) I strains of *C. elegans* were obtained from the Caenorhabditis Genetics Center at the University of Minnesota (CGC, Minneapolis, MN, USA). The N2 strain was kindly provided by Dr Martin J. Simard from Laval University, Cancer Research Center, Vancouver, BC, Canada.

### 2.3. Fat Accumulation Analysis

#### 2.3.1. Oil Red O Staining

The Oil Red O staining protocol was performed according to a procedure previously described [39]. In brief, 3-day-old synchronized worms fed OP50, probiotics, or OP50 with lipase inhibitor orlistat (Sigma-Aldrich, Saint-Louis, MO, USA) were fixed in an isopropanol solution and stained immediately for 18 h in an Oil Red O solution (Sigma-Aldrich, Saint-Louis, MO, USA). After stain solution removal, the worms were kept in 0.01% M9-tritron X100 (Sigma-Aldrich, Saint-Louis, MO, USA) until microscope observation and image capture at 10X magnification (LMC4 and Sebacam10C, Laxcoinc, Mill Creek, WA, USA). Oil Red O intensity was estimated using ImageJ software version 1.54i (NIH, Bethesda, MD, USA).

#### 2.3.2. Nile Red Staining

The Nile Red staining protocol was performed according to the assays described previously for probiotics [36]. Synchronized nematodes were cultured until the young adult stage and then transferred to plates containing various types of food and Nile Red (Sigma–Aldrich, Saint-Louis, MO, USA) at a concentration of 0.05 µg/mL. Three-day-old worms fed OP50, probiotics, or OP50 with control with a lipase inhibitor orlistat (Sigma-Aldrich, Saint-Louis, MO, USA) have been tested. After 3 days, the nematodes were collected, washed, and maintained in M9, and the fluorescence intensity was measured using a fluorometer (Discovery, Promega, Madison, WI, USA) with an excitation λ of 480 nm and an emission λ of 571 nm.

### 2.4. Lifespan Analysis

The lifespan of *C. elegans* was measured using synchronized worms. The wild-type strain (N2) and the mutant strains daf-16 (GR1307), daf-2 (CB1370), and pmk-1 (KU25) at 20 °C have been used. The assay consists of monitoring the mortality of a worm population in a specific condition. Synchronized nematodes were cultured until the young adult stage on OP50 and then transferred to plates containing various types of food. A total of 20–30 worms were added to plates and 3 plates were used per condition. The plates were scored and counted, and surviving worms were transferred every 2 days to a fresh plate with the same food until the end of the experiment, when no living worms remained. For some experiments, 2% of the final glucose concentration was added to the Nematode Growth Media (NGM) standard plate and dried for 24 h under a sterile hood. Statistical analysis was performed using OASIS2 https://sbi.postech.ac.kr/oasis2 (accessed on 17 May 2022) according to the procedure described; a Log-rank test analysis was performed with the mean lifespan calculation, Bonferroni-corrected *p*-value, and percentage of change compared to the control.

### 2.5. Oxidative Stress Assay

Synchronized worms, fed OP50 until they reached young adulthood, were transferred to plates supplemented with probiotics or OP50. After 3 days, in 96-well plates (5–7 worms per well), the worms were incubated at 20 °C for 6 h. After 3 days, H_2_O_2_ solutions (1.5 mM, 3 mM, and 5 mM) were prepared in distilled water. The worms were observed and scored for lethality under an optic microscope (SMZ-140, Motic Instruments, Richmond, VA, Canada) after 6 h of treatment.

## 3. Results

### 3.1. L. acidophilus CL1285 Reduces Fat Content and Exerts Multiple Beneficial Effects in a C. elegans Model

To assess whether probiotic feeding influences fat accumulation in a worm model, two stains were used, a fixative-based stain (Oil Red O) and a live stain (Nile Red); these two common lipid stains have been used to evaluate lipid distribution in various tissues and models. These methods allow for the visualization of fat content by microscopy (Oil Red O) and a low-throughput fat mass score can be determined using ImageJ [39]; Nile Red enables the fluorescence measurement of one hundred worms under native conditions, independent of image capture, and these measurements can be controlled with orlistat, a hypolipidemic drug [35]. At the same time, this dual approach may also strengthen results due to certain discrepancies between fixed and live imaging methods.

**Figure 1:**
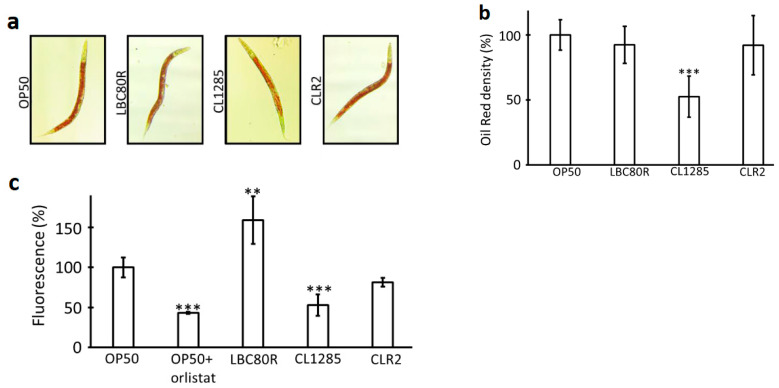
Reduction in fat accumulation in *C. elegans* fed with CL1285. (**a**) Oil Red visualization of N2 worms fed various bacteria for 3 days after reaching adult stage. (**b**) Oil Red density estimation using ImageJ quantification of 10 representative Oil-Red-stained worms; 100% is fixed for N2 worms given OP50 food. *** *p* < 0.05; one way-ANOVA with Tukey’s HSD test relative to OP50 control. (**c**) Fluorescence measurement of Nile-Red-stained worms (approximately 180 worms, in triplicate) after 3 days of being fed various sources of food. Orlistat was used as a control inhibitor of lipid accumulation; 100% is fixed for N2 worms given OP50 food. *** *p* < 0.01; ** *p* < 0.05; *t* test relative to the OP50 control.

Synchronized worms at the adult stage were grown on NGM plates, seeded with fresh cultures of OP50, *L. acidophilus* CL1285, *L. casei* LBC80R, or *L. rhamnosus* at 20 °C and observed under a microscope. No morphological difference was detected until 3 days of exposure, except for worms fed CL1285, which appeared thinner than those fed the other probiotics or the control group. Oil Red O staining using the isopropanol fixation method was performed to evaluate fat accumulation and distribution; the results revealed no difference when comparing OP50 administration to *L. casei* LBC80R and *L. rhamnosus* administration, but the staining in *L. acidophilus* CL1285-fed worms appeared weaker (Figure 1a). Using ImageJ, measurements of 10 representative worms revealed a significant difference between worms fed OP50 or CL1285, with an approximate 50% reduction in intensity (Figure 1b). The intensity in LBC80R- and CLR2-fed worms were similar to those of the OP50 control. In parallel, Nile Red staining followed by fluorescence measurements after 3 days of adulthood revealed differences (Figure 1c). Clearly, LBC80R consumption increased the fluorescence signal, indicating an increase in fatty acids, while CLR-2 displayed a weaker signal than the OP50 and orlistat control worms. However, CL1285-fed worms exhibited a significant decrease in fluorescence, similar to the results of previous fixative staining (Figure 1a,b). These observations confirmed that CL1285 could attenuate fat deposition in *C. elegans* via both methods.

**Figure 2:**
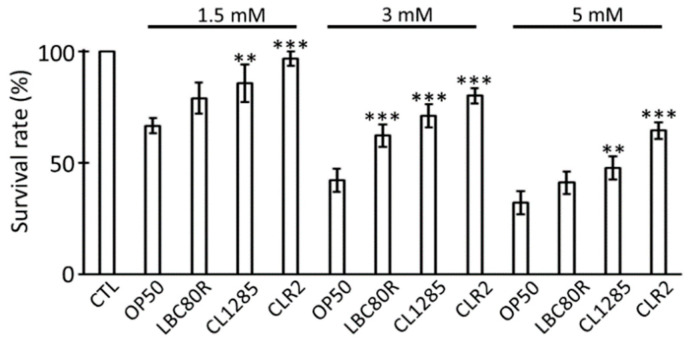
Oxidative stress protection of probiotic bacteria in *C. elegans*: H_2_O_2_ survival assay on worms fed different food for 3 days after adult stage and 6 h on H_2_O_2_ (1.5, 3 and 5 mM); 100% is fixed for untreated worms given OP50. *** *p* < 0.01; ** *p* < 0.05; one way-ANOVA with Tukey’s HSD test relative to OP50 control.

Afterward, probiotics were evaluated for their ability to protect against oxidative stress. Many procedures have been described in the literature, and various oxidative stress molecules are used; herein, H_2_O_2_ was selected to trigger stress because of its safety, rapid effect, and ease of use [35]. At 3 days of adulthood, worms fed with OP50 or probiotics were treated for 6 h with 1.5, 3, or 5 mM H_2_O_2_ solution, and viability was scored after exposure. At all concentrations tested, the presence of the probiotic partially protected the nematodes against H_2_O_2_ exposure in a dose-dependent manner (Figure 2). Exposure to concentrations of 1.5 mM and 3 mM was more effective for evaluating protection, while a concentration of 5 mM was too harmful. However, CLR2 remained the probiotic candidate that offered the most protection at all H_2_O_2_ concentrations. Overall resistance was observed in all test conditions when compared to OP50-fed worms. In fact, CLR-2 displayed the greatest protective effect, while LBC80R offered limited protection and CL1285 had intermediate protection (Figure 2).

**Figure 3:**
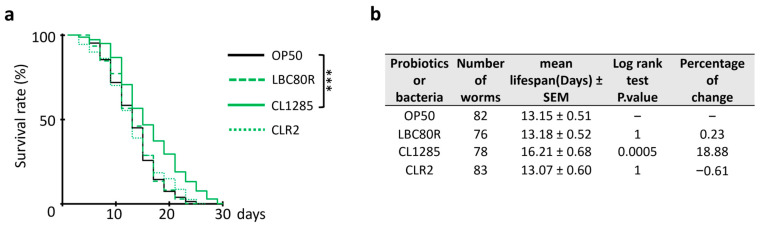
Life extension of *C. elegans* fed CL1285 probiotic. (**a**) Survival curve and table of N2 worms given various bacterial expositions after the adult stage. *** indicates the results of Log-rank test analysis (*p* < 0.01) performed with OASIS2 online software (accessed on 17 May 2022). (**b**) The table describes number of worms, their mean lifespan, the log-rank test Bonferroni-corrected *p*-value, and the percentage of change compared to control. Three independent experiments were performed.

With these interesting data regarding fat accumulation and partial resistance to oxidative stress, we assumed that CL1285 and/or CLR2 could extend worm lifespan, as other probiotics with one of these features increased longevity [33,34,35,36,40]. To evaluate worm lifespan, the mortality rate of worms exposed to various probiotics once they reached the adult stage was scored. CL1285 clearly promoted longevity under these conditions (Figure 3a) and extended the mean lifespan to approximately 3 days (Figure 3b), while worms fed the CLR-2 strain, which exhibited the greatest oxidative resistance to H_2_O_2_ and a reduced fat content, did not have a prolonged lifespan. Although LBC80R was less protective against oxidative stress than other probiotic strains and it increased fat in one of the two methods used, no long-term deleterious effect on lifespan was noted. Taken together, these results indicate that CL1285 could be of interest in the context of obesity and metabolic syndrome.

### 3.2. L. acidophilus CL1285 Limits Fat Reduction and Increases Lifespan via a Glucose/daf-16-Dependent Pathway

**Figure 4:**
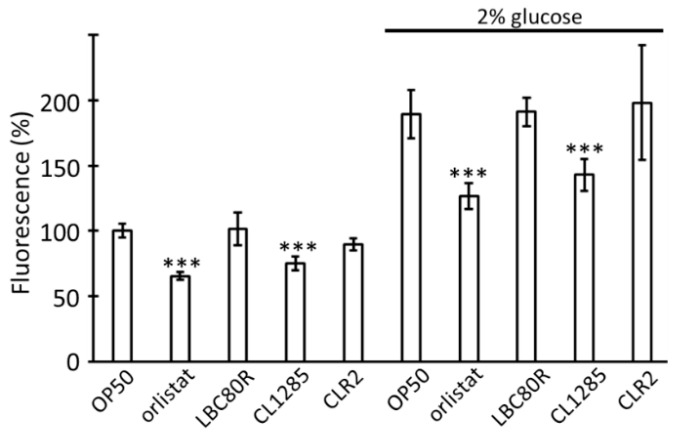
Glucose limits fat reduction in CL1285. Fluorescence measurement of Nile Red stained worms (approximately 180 worms, in triplicate) after 3 days with various sources food with or without 2% glucose addition in NGM media. Orlistat was used as a control inhibitor of lipid accumulation; 100% is fixed for N2 given OP50 food without glucose. *** *p* < 0.05; one way-ANOVA with Tukey’s HSD test relative to each OP50 control.

The addition of glucose is known to reduce lifespan and increase fat content in *C. elegans* [41,42]; therefore, this condition could be considered similar to obesity and metabolic syndrome in humans [43]. To estimate the capacity of CL1285 to counteract the glucose enrichment effect, a fat-reduction test in 2% enriched NGM media was performed using Nile Red staining as previously described; CL1285 could still limit fat accumulation under these particular conditions (Figure 4). Although glucose addition triggered an overall increase in fluorescence, CL1285-fed worms exhibited a fluorescence level comparable to that of the OP50 control worms and reached the level observed in orlistat-drug-treated worms in glucose-enriched media. LBC80R and CLR2 were unable to limit fat accumulation in glucose-enriched media (Figure 4). In this second set of Nile Red measurements, LBC80R- and CLR2-treated worms exhibited significant variability in fluorescence compared to that in the first experiment, with a loss of phenotypes observed in the first experiment (Figure 1c); this variability was repeatedly observed. However, CL1285 still significantly reduced fat accumulation by approximately 25%. This reduction was conserved in glucose-enriched media when compared to that of the OP50 control (26%). 

**Figure 5:**
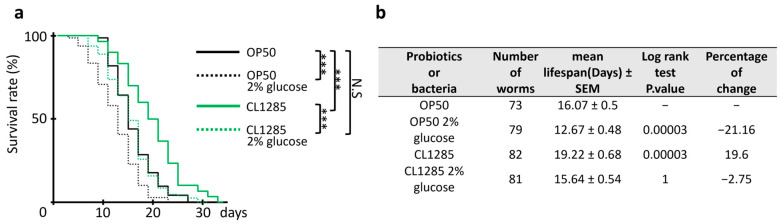
Glucose limits the benefits of CL1285 in lifespan. (**a**) Survival curve of N2 worms given various bacterial expositions after reaching the adult stage. *** *p* < 0.001 and N.S (not significant) indicates the results of log-rank test analysis performed with OASIS2 online software. (**b**) The table describes the mean lifespan, the log-rank test Bonferroni-corrected *p*-value, and the percentage of change compared to control.

After these results were obtained, the effect of CL1285 on the lifespan of worms was evaluated over time and compared to the effect of OP50 under the same conditions (Figure 5a). In line with the previous fat accumulation results, glucose administration to CL1285-fed worms limited the lifespan extension to that of OP50-fed worms without glucose addition, but the difference between the OP50 control and CL1285 under glucose-enriched conditions remained significant. An approximate 3-day mean lifespan distinguished the control and CL1285 strains in either regular or glucose-enriched media (Figure 5b). 

**Figure 6:**
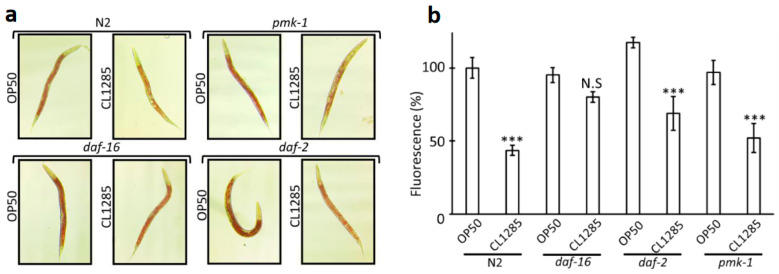
Mechanistic study of CL1285 fat reduction using *C. elegans* mutant strains. (**a**) Oil Red visualization of representative N2 worms or mutant line fed OP50 or CL1285 for 3 days after reaching adult stage. (**b**) Fluorescence measurement of Nile Red stained worms (approximately 180 worms, in triplicate) after being fed various sources of food for 3 days. Orlistat was used as the control inhibitor of lipid accumulation; 100% is fixed for N2 given OP50 food. *** *p* < 0.01; one way-ANOVA with Tukey’s HSD test relative to OP50 control for each mutant strain. N.S (not significant).

Because glucose could partially counteract the protective effect of CL1285 on lifespan and fat accumulation [41], we hypothesized that CL1285 interacts with the glucose signaling pathway. Based on the results of a recent review [44], and to investigate the signaling pathways that CL1285 influences, three mutants were chosen, with a focus on lifespan and lipid accumulation: *daf-2*, *daf-16*, and *pmk-1.* The *daf-2* mutant CB1370 is an insulin/IGF-1 pathway mutant without an insulin-like receptor that regulates daf-16 phosphorylation and translocation to the nucleus, resulting in an extended lifespan. The *daf-16* mutant GR1307, a well-described and well-studied line with a reduced lifespan and insensitivity to glucose [41], lacks the gene encoding the daf-16 forkhead box transcription factor, which is evolutionarily conserved and functions downstream of daf-2 receptor activation. *pmk*-1 KU-25 is defective in the p38 homolog PMK-1, which is a terminal MAPK of a stress/immune signaling pathway involved in the lifespan extension effects of various probiotics. Oxidative stress triggers the activation of the p38 MAPK pathway, which regulates the nuclear localization of the transcription factor SKN-1 by phosphorylation [45]. Interestingly, CL1285 led to significant fat reduction in the *daf-2* and *pmk-1* mutants compared to OP50 in Oil Red (Figure 6a) and in Nile Red (Figure 6b). In the Nile Red fluorescence measurement, the fat reduction in *daf-2* and *pmk-1* was similar to that in the N2 control, with a decrease of around 40 to 50 percent (Figure 6b). The decrease in the *daf-16* mutant was not significant and was approximatively 10% less than the OP50 control; these results showed that the daf-16 gene product is required for CL1285 to impact fat reduction because in a strain with this genetic background, fat reduction was abolished. 

**Figure 7:**
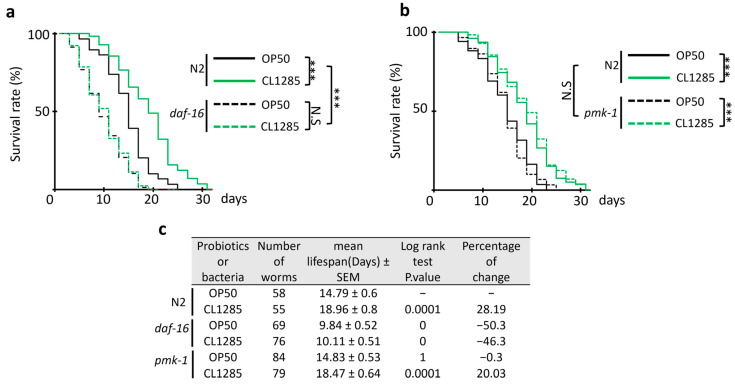
Mechanistic study of life extension of CL1285 using *C. elegans* mutant strains. Survival curve of worms given various bacterial expositions after adult stage using the *C. elegans* mutant strains *daf-16* (**a**) and *pmk-1* (**b**). *** *p* < 0.01 and N.S (not significant) indicates the results of log-rank test analysis performed with OASIS2 online software. (**c**) The table describes the mean lifespan, the log-rank test Bonferroni-corrected *p*-value, and the percentage of change compared to control.

To confirm that lifespan extension conferred by CL1285 is mediated by *daf-16* activity, a lifespan experiment was performed using *daf-16*, *pmk-1,* and OP50. The experiment clearly demonstrated no difference in lifespan when comparing OP50 to CL1285 in the *daf-16* mutant worms (Figure 7a), and CL1285 did not extend lifespan. In contrast to the *daf-16* line, the *pmk-1* and N2 control lines show similar lifespan extensions (Figure 7b). *daf-2* was partially evaluated, but lifespan measurement was not completed due to the extended lifespan of this strain, partial dauer formation, and developmental delay; however, there were no differences between OP50- and CL1285-fed worms in the first 25 days of the experiment. Similar to the action on fat accumulation, the daf-16 gene product is required for lifespan extension because the absence of this gene abolishes the benefits triggered by CL1285.

**Figure 8:**
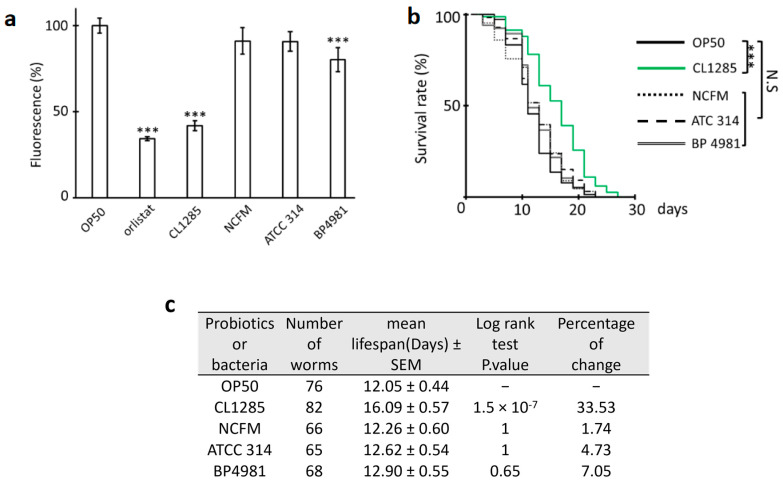
Comparative study of life extension and fat reduction in CL1285 and commercial *L. acidophilus* strains. (**a**) Fluorescence measurement of Nile Red stained worms (approximately 180 worms, in triplicate) after being fed with various sources of food for 3 days. Orlistat was used as a control inhibitor of lipid accumulation; 100% is fixed for N2 given OP50 food. *** *p* < 0.01; one-way-ANOVA with Tukey’s HSD test relative to OP50 control. (**b**) Survival curve of N2 worms given various bacterial expositions after adult stage using the *C. elegans* N2 strain. *** *p* < 0.01 and N.S (not significant) indicates the results of log-rank test analysis performed with OASIS2 online software. (**c**) The table describes the mean lifespan, the log-rank test Bonferroni-corrected *p*-value, and the percentage of change compared to control.

Taken together, these results showed that the daf-16 gene product is required for CL1285 fat metabolism and lifespan extension, and these effects are glucose-dependent. With the aim of determining whether the benefits of CL1285 in *C. elegans* are strain-specific, three commercially available strains were tested in fat reduction and lifespan experiments, and the obtained results were compared to those of CL1285 (Figure 8). Nile Red measurement of worms fed the *L. acidophilus* strains NCFM, ATCC 314, and BP4981 were analyzed and compared to that of CL1285 (Figure 8a). First, the Nile Red fluorescence measurements showed an overall reduction in fat accumulation in 3-day-old adult worms fed NCFM or ATCC 314; however, this reduction was only significant with BP4981 when compared to OP50, which showed 20% less fluorescence. However, the reduction in the fat accumulation of CL1285-fed worms was more efficient with a 60% decrease in fluorescence intensity, demonstrating that this is a unique property of this strain (Figure 8a). Regarding lifespan, none of the strains tested increased the worm lifespan, aside from CL1285 (Figure 8b,c).

## 4. Discussion

The proverbial saying “you are what you eat” has been known for decades, and the association between diet and life expectancy indicates a connection between good health and eating well. More scientific evidence has recently emerged on this topic, and this evidence broadens this concept by including more habits that are essential for maintaining health and protecting against diseases such as cardiovascular disease and cancer [46]. These habits include adopting an active lifestyle and reducing the consumption of toxic substances such as tobacco and alcohol. The link between food quality, quantity, and lifespan is obvious and not specific to *C. elegans*. Generally, the intake of highly caloric food with a low intake of fiber and fermented foods leads to body fat accumulation, mediated by hyperglycemia, hyperlipidemia, and insulin-resistance mechanisms, which contribute to hyperactive stimulation of intracellular biochemical pathways with increased ROS production and free radicals; these effects have a detrimental effect on cellular aging, innate immunity, and protection against environmental stress. As a result, the duration of life is affected. Previous studies have used worms as model organisms to efficiently screen *Lactobacillus* spp. or other potential probiotic strains for specific and beneficial activities [34,35,36,40,43]. Here, we described a strain that induces fat reduction under normal and glucose-enriched conditions, extends lifespan, and protects against oxidative stress. These results demonstrated that the fat reduction and lifespan extension conferred by CL1285 are genetically dependent on the daf-16 gene. In fact, the DAF-16/FOXO transcription factor integrates signals from many pathways, such as insulin/IGF-1, JNK, AMPK, TOR, and germline signaling [47], and can thus be considered a central signal reception platform. Alongside DAF-16/FOXO, SKN-1 and HSF-1 which are two transcription factors are under daf-2 regulation; these are involved, respectively, in xenobiotic/oxidative stress responses and in heat-shock protein response [48]. SKN-1, modulated by p38-MAPK, is a negative regulator of DAF-16 and have a complex interplay connecting insulin signaling, cellular metabolism, and oxidative stress resistance [49,50,51]. A transcriptional study of insulin-signaling-associated transcription factors revealed that daf-16 plays a central regulatory role and revealed that, although this transcription factor is essentially an activator, it acts at the complex, genome-wide level, and is a major determining factor [52]. Longevity, fat storage, dauer formation, and stress resistance are among the main mechanisms that are regulated by daf-16 via the insulin signaling pathway [53]; our studies showed that CL1285 modulates the first two biological processes described. In this study, we did not explore dauer formation or stress resistance. Although oxidative stress induced by H_2_O_2_ was not different between the N2 and mutant lines, we cannot draw conclusions about general stress resistance because only ROS were generated by H_2_O_2_ induction. This rapid assay did not allow us to determine whether CL1285 protection is mediated by daf-16. Moreover, this mutant line displays greater stress resistance because *daf-16* at least partially regulates genes involved in the stress response [54,55].

By acting via the daf-16 pathway, in this study, we demonstrated that the probiotic CL1285 anchored health benefits to the daf-16 pathway; and this effect was strain-specific. Nevertheless, more in-depth and extensive research is needed to establish a clear mechanism of action. Data from other groups indicate that CL1285 could act on fat metabolism, similar to *Pediococcus acidilactici* CECT9879 [43]. In a *C. elegans* model, this strain inhibited fatty acid (FA) biosynthesis and activated mitochondrial and peroxisomal FA degradation. CL1285 also reduced ROS levels and prolonged worm lifespan in a glucose-enriched medium. The extension of worm lifespan by CL1285 is greater in the standard condition; meanwhile, under glucose-enriched conditions, the lifespan extension by CL1285 was reduced to a normal duration. An explanation of this effect could be that glucose counteracts the CL1285-mediated increase in lifespan by a mechanism that does not lead to mRNA daf-16 overexpression and/or protein production in glucose-enriched media. Another very interesting study demonstrated that bacterial processing of glucose should be carefully considered when studying the lifespan of *C. elegans* [56]. CL1285 glucose processing could lead to detrimental effects that were not apparent under normal growth conditions. However, in this model, the fat-lowering properties were apparent in all mutants used and in all the experiments performed, with or without glucose supplementation, except with the *daf-16* mutant. In the *daf-2* mutant, the fat-lowering effect clearly indicated that the benefits of CL1285 are daf-2-independent, indicating that a mechanism independent of insulin signaling is involved. This mechanism should be investigated further with various mutants, qPCR, or transcriptomic strategies to target genes involved in fat metabolism and daf-16-dependent genes.

Regarding the strain properties, our group published a recent paper that could explain the fat accumulation in the *C. elegans* model: *L. acidophilus* CL1285 has feruloyl esterase activity, bile salt hydrolase enzymes, and cholesterol assimilation properties [57]. Moreover, the impact of CL1285 was previously evaluated in a hypercholesterolemic hamster model, and the results showed that animals supplemented with this probiotic had a decreased level of non-HDLC (*p* = 0.08) and an increased HDL/cholesterol ratio (*p* ≤ 0.05) (unpublished data). Unfortunately, other metabolic or physical changes, such as weight loss, were not visible under these experimental conditions. The C. elegans model’s conclusion suggests that it may be critical to use a high-sugar diet instead of a high-fat diet in the future for hypercholesterolemia study in mammals. This difference in results obtained with *C. elegans* and hamster for fat reduction may be due to other limitations, such as a poor yield resulting from bacterial lyophilization, an inadequate animal model, and/or inadequate food distribution.

## 5. Conclusions

In conclusion, our data indicate that *L. acidophilus* CL1285 had a beneficial probiotic effect on fat accumulation, lifespan, and oxidative stress in a *C. elegans* model and these properties are specific to CL1285 and are not shared with common commercially available *L. acidophilus* strains. Furthermore, the daf-16 gene product seems to be needed for the fat and lifespan phenotypes, and experiments performed under glucose-enriched conditions indicate that this strain could be applied for metabolic syndromes. Further research is needed to elucidate the mechanism in *C. elegans* and determine the origin of the active molecules or metabolites in CL1285, and appropriate animal studies should also be performed before this probiotic of interest can be evaluated in humans.

## Data Availability

Data are contained within the article.

## References

[1] Thursby E., Juge N. (2017). Introduction to the human gut microbiota. Biochem. J..

[2] Rowland I., Gibson G., Heinken A., Scott K., Swann J., Thiele I., Tuohy K. (2018). Gut microbiota functions: Metabolism of nutrients and other food components. Eur. J. Nutr..

[3] The Human Microbiome Project Consortium (2012). Structure, function and diversity of the healthy human microbiome. Nature.

[4] Scheithauer T.P.M., Rampanelli E., Nieuwdorp M., Vallance B.A., Verchere C.B., van Raalte D.H., Herrema H. (2020). Gut Microbiota as a Trigger for Metabolic Inflammation in Obesity and Type 2 Diabetes. Front. Immunol..

[5] Kinashi Y., Hase K. (2021). Partners in Leaky Gut Syndrome: Intestinal Dysbiosis and Autoimmunity. Front. Immunol..

[6] Lobionda S., Sittipo P., Kwon H.Y., Lee Y.K. (2019). The Role of Gut Microbiota in Intestinal Inflammation with Respect to Diet and Extrinsic Stressors. Microorganisms.

[7] Butaye P., Devriese L.A., Haesebrouck F. (2003). Antimicrobial growth promoters used in animal feed: Effects of less well known antibiotics on gram-positive bacteria. Clin. Microbiol. Rev..

[8] Everard A., Belzer C., Geurts L., Ouwerkerk J.P., Druart C., Bindels L.B., Guiot Y., Derrien M., Muccioli G.G., Delzenne N.M. (2013). Cross-talk between Akkermansia muciniphila and intestinal epithelium controls diet-induced obesity. Proc. Natl. Acad. Sci. USA.

[9] Derrien M., Vaughan E.E., Plugge C.M., de Vos W.M. (2004). Akkermansia muciniphila gen. nov., sp. nov., a human intestinal mucin-degrading bacterium. Int. J. Syst. Evol. Microbiol..

[10] Hill C., Guarner F., Reid G., Gibson G.R., Merenstein D.J., Pot B., Morelli L., Canani R.B., Flint H.J., Salminen S. (2014). Expert consensus document. The International Scientific Association for Probiotics and Prebiotics consensus statement on the scope and appropriate use of the term probiotic. Nat. Rev. Gastroenterol. Hepatol..

[11] Zawistowska-Rojek A., Tyski S. (2018). Are Probiotic Really Safe for Humans?. Pol. J. Microbiol..

[12] Ritchie M.L., Romanuk T.N. (2012). A meta-analysis of probiotic efficacy for gastrointestinal diseases. PLoS ONE.

[13] Ahmad K., Fatemeh F., Mehri N., Maryam S. (2013). Probiotics for the treatment of pediatric helicobacter pylori infection: A randomized double blind clinical trial. Iran. J. Pediatr..

[14] Armuzzi A., Cremonini F., Bartolozzi F., Canducci F., Candelli M., Ojetti V., Cammarota G., Anti M., De Lorenzo A., Pola P. (2001). The effect of oral administration of Lactobacillus GG on antibiotic-associated gastrointestinal side-effects during Helicobacter pylori eradication therapy. Aliment. Pharmacol. Ther..

[15] Dicks L.M., ten Doeschate K. (2010). Enterococcus mundtii ST4SA and Lactobacillus plantarum 423 alleviated symptoms of Salmonella infection, as determined in Wistar rats challenged with Salmonella enterica serovar Typhimurium. Curr. Microbiol..

[16] Silva A.M., Barbosa F.H., Duarte R., Vieira L.Q., Arantes R.M., Nicoli J.R. (2004). Effect of Bifidobacterium longum ingestion on experimental salmonellosis in mice. J. Appl. Microbiol..

[17] Gill H.S., Shu Q., Lin H., Rutherfurd K.J., Cross M.L. (2001). Protection against translocating Salmonella typhimurium infection in mice by feeding the immuno-enhancing probiotic Lactobacillus rhamnosus strain HN001. Med. Microbiol. Immunol..

[18] Homayouni A., Bastani P., Ziyadi S., Mohammad-Alizadeh-Charandabi S., Ghalibaf M., Mortazavian A.M., Mehrabany E.V. (2014). Effects of probiotics on the recurrence of bacterial vaginosis: A review. J. Low. Genit. Tract. Dis..

[19] Wang Y., Li X., Ge T., Xiao Y., Liao Y., Cui Y., Zhang Y., Ho W., Yu G., Zhang T. (2016). Probiotics for prevention and treatment of respiratory tract infections in children: A systematic review and meta-analysis of randomized controlled trials. Medicine.

[20] Bermudez-Brito M., Plaza-Díaz J., Muñoz-Quezada S., Gómez-Llorente C., Gil A. (2012). Probiotic mechanisms of action. Ann. Nutr. Metab..

[21] Balta I., Butucel E., Mohylyuk V., Criste A., Dezmirean D.S., Stef L., Pet I., Corcionivoschi N. (2021). Novel Insights into the Role of Probiotics in Respiratory Infections, Allergies, Cancer, and Neurological Abnormalities. Diseases.

[22] Gao X.W., Mubasher M., Fang C.Y., Reifer C., Miller L.E. (2010). Dose-response efficacy of a proprietary probiotic formula of Lactobacillus acidophilus CL1285 and Lactobacillus casei LBC80R for antibiotic-associated diarrhea and Clostridium difficile-associated diarrhea prophylaxis in adult patients. Am. J. Gastroenterol..

[23] Gunaratnam S., Diarra C., Paquette P.D., Ship N., Millette M., Lacroix M. (2021). The Acid-Dependent and Independent Effects of Lactobacillus acidophilus CL1285, Lacticaseibacillus casei LBC80R, and Lacticaseibacillus rhamnosus CLR2 on Clostridioides difficile R20291. Probiotics Antimicrob. Proteins.

[24] Park D.Y., Ahn Y.T., Park S.H., Huh C.S., Yoo S.R., Yu R., Sung M.K., McGregor R.A., Choi M.S. (2013). Supplementation of Lactobacillus curvatus HY7601 and Lactobacillus plantarum KY1032 in diet-induced obese mice is associated with gut microbial changes and reduction in obesity. PLoS ONE.

[25] Song W., Song C., Li L., Wang T., Hu J., Zhu L., Yue T. (2021). Lactobacillus alleviated obesity induced by high-fat diet in mice. J. Food Sci..

[26] Chen Y.T., Chiou S.Y., Hsu A.H., Lin Y.C., Lin J.S. (2022). Lactobacillus rhamnosus Strain LRH05 Intervention Ameliorated Body Weight Gain and Adipose Inflammation via Modulating the Gut Microbiota in High-Fat Diet-Induced Obese Mice. Mol. Nutr. Food Res..

[27] Ferrarese R., Ceresola E.R., Preti A., Canducci F. (2018). Probiotics, prebiotics and synbiotics for weight loss and metabolic syndrome in the microbiome era. Eur. Rev. Med. Pharmacol. Sci..

[28] Abenavoli L., Scarpellini E., Colica C., Boccuto L., Salehi B., Sharifi-Rad J., Aiello V., Romano B., De Lorenzo A., Izzo A.A. (2019). Gut Microbiota and Obesity: A Role for Probiotics. Nutrients.

[29] Sanchez M., Darimont C., Panahi S., Drapeau V., Marette A., Taylor V.H., Doré J., Tremblay A. (2017). Effects of a Diet-Based Weight-Reducing Program with Probiotic Supplementation on Satiety Efficiency, Eating Behaviour Traits, and Psychosocial Behaviours in Obese Individuals. Nutrients.

[30] Kadooka Y., Sato M., Imaizumi K., Ogawa A., Ikuyama K., Akai Y., Okano M., Kagoshima M., Tsuchida T. (2010). Regulation of abdominal adiposity by probiotics (Lactobacillus gasseri SBT2055) in adults with obese tendencies in a randomized controlled trial. Eur. J. Clin. Nutr..

[31] Gomes A.C., de Sousa R.G., Botelho P.B., Gomes T.L., Prada P.O., Mota J.F. (2017). The additional effects of a probiotic mix on abdominal adiposity and antioxidant Status: A double-blind, randomized trial. Obesity.

[32] Kwon G., Lee J., Lim Y.H. (2016). Dairy Propionibacterium extends the mean lifespan of *Caenorhabditis elegans* via activation of the innate immune system. Sci. Rep..

[33] Li W., Gao L., Huang W., Ma Y., Muhammad I., Hanif A., Ding Z., Guo X. (2022). Antioxidant properties of lactic acid bacteria isolated from traditional fermented yak milk and their probiotic effects on the oxidative senescence of *Caenorhabditis elegans*. Food Funct..

[34] Hu R., Zhang Y., Qian W., Leng Y., Long Y., Liu X., Li J., Wan X., Wei X. (2022). Pediococcus acidilactici Promotes the Longevity of *C. elegans* by Regulating the Insulin/IGF-1 and JNK/MAPK Signaling, Fat Accumulation and Chloride Ion. Front. Nutr..

[35] Grompone G., Martorell P., Llopis S., González N., Genovés S., Mulet A.P., Fernández-Calero T., Tiscornia I., Bollati-Fogolín M., Chambaud I. (2012). Anti-inflammatory Lactobacillus rhamnosus CNCM I-3690 strain protects against oxidative stress and increases lifespan in *Caenorhabditis elegans*. PLoS ONE.

[36] Martorell P., Llopis S., González N., Chenoll E., López-Carreras N., Aleixandre A., Chen Y., Karoly E.D., Ramón D., Genovés S. (2016). Probiotic Strain Bifidobacterium animalis subsp. lactis CECT 8145 Reduces Fat Content and Modulates Lipid Metabolism and Antioxidant Response in *Caenorhabditis elegans*. J. Agric. Food Chem..

[37] Savini I., Catani M.V., Evangelista D., Gasperi V., Avigliano L. (2013). Obesity-associated oxidative stress: Strategies finalized to improve redox state. Int. J. Mol. Sci..

[38] Wang K., Chen S., Zhang C., Huang J., Wu J., Zhou H., Jin L., Qian X., Jin J., Lyu J. (2018). Enhanced ROS production leads to excessive fat accumulation through DAF-16 in *Caenorhabditis elegans*. Exp. Gerontol..

[39] Wählby C., Conery A.L., Bray M.A., Kamentsky L., Larkins-Ford J., Sokolnicki K.L., Veneskey M., Michaels K., Carpenter A.E., O’Rourke E.J. (2014). High- and low-throughput scoring of fat mass and body fat distribution in *C. elegans*. Methods.

[40] Kumar A., Joishy T., Das S., Kalita M.C., Mukherjee A.K., Khan M.R. (2022). A Potential Probiotic Lactobacillus plantarum JBC5 Improves Longevity and Healthy Aging by Modulating Antioxidative, Innate Immunity and Serotonin-Signaling Pathways in *Caenorhabditis elegans*. Antioxidants.

[41] Lee S.J., Murphy C.T., Kenyon C. (2009). Glucose shortens the life span of *C. elegans* by downregulating DAF-16/FOXO activity and aquaporin gene expression. Cell Metab..

[42] Garcia A.M., Ladage M.L., Dumesnil D.R., Zaman K., Shulaev V., Azad R.K., Padilla P.A. (2015). Glucose induces sensitivity to oxygen deprivation and modulates insulin/IGF-1 signaling and lipid biosynthesis in *Caenorhabditis elegans*. Genetics.

[43] Yavorov-Dayliev D., Milagro F.I., Ayo J., Oneca M., Aranaz P. (2022). Pediococcus acidilactici CECT9879 (pA1c) Counteracts the Effect of a High-Glucose Exposure in *C. elegans* by Affecting the Insulin Signaling Pathway (IIS). Int. J. Mol. Sci..

[44] Tsai Y.C., Cheng L.H., Liu Y.W., Jeng O.J., Lee Y.K. (2021). Gerobiotics: Probiotics targeting fundamental aging processes. Biosci. Microbiota Food Health.

[45] Inoue H., Hisamoto N., An J.H., Oliveira R.P., Nishida E., Blackwell T.K., Matsumoto K. (2005). The *C. elegans* p38 MAPK pathway regulates nuclear localization of the transcription factor SKN-1 in oxidative stress response. Genes Dev..

[46] Knoops K.T., de Groot L.C., Kromhout D., Perrin A.E., Moreiras-Varela O., Menotti A., van Staveren W.A. (2004). Mediterranean diet, lifestyle factors, and 10-year mortality in elderly European men and women: The HALE project. JAMA.

[47] Sun X., Chen W.D., Wang Y.D. (2017). DAF-16/FOXO Transcription Factor in Aging and Longevity. Front. Pharmacol..

[48] Koch K., Havermann S., Büchter C., Wätjen W. (2014). *Caenorhabditis elegans* as model system in pharmacology and toxicology: Effects of flavonoids on redox-sensitive signalling pathways and ageing. Sci. World J..

[49] Blackwell T.K., Steinbaugh M.J., Hourihan J.M., Ewald C.Y., Isik M. (2015). SKN-1/Nrf, stress responses, and aging in *Caenorhabditis elegans*. Free Radic. Biol. Med..

[50] Deng J., Dai Y., Tang H., Pang S. (2020). SKN-1 Is a Negative Regulator of DAF-16 and Somatic Stress Resistance in *Caenorhabditis elegans*. G3.

[51] Phan H.D., Nguyen T.T.M., Lee S., Seo M., An Y.J., de Guzman A.C.V. (2023). The metabolic contribution of SKN-1/Nrf2 to the lifespan of *Caenorhabditis elegans*. Metabolomics.

[52] Kaushik N., Rastogi S., Verma S., Pandey D., Halder A., Mukhopadhyay A., Kumar N. (2021). Transcriptome Analysis of Insulin Signaling-Associated Transcription Factors in *C. elegans* Reveal Their Genome-Wide Target Genes Specificity and Complexity. Int. J. Mol. Sci..

[53] Tissenbaum H.A. (2018). DAF-16: FOXO in the Context of *C. elegans*. Curr. Top. Dev. Biol..

[54] Weinkove D., Halstead J.R., Gems D., Divecha N. (2006). Long-term starvation and ageing induce AGE-1/PI 3-kinase-dependent translocation of DAF-16/FOXO to the cytoplasm. BMC Biol..

[55] Wu Y., Masurat F., Preis J., Bringmann H. (2018). Sleep Counteracts Aging Phenotypes to Survive Starvation-Induced Developmental Arrest in *C. elegans*. Curr. Biol..

[56] Kingsley S.F., Seo Y., Allen C., Ghanta K.S., Finkel S., Tissenbaum H.A. (2021). Bacterial processing of glucose modulates *C. elegans* lifespan and healthspan. Sci. Rep..

[57] Frappier M., Auclair J., Bouasker S., Gunaratnam S., Diarra C., Millette M. (2022). Screening and Characterization of Some Lactobacillaceae for Detection of Cholesterol-Lowering Activities. Probiotics Antimicrob. Proteins.

